# Detection, identification and quantification of NGTs in EU authorization procedures – No solution without legislative change

**DOI:** 10.12688/openreseurope.23629.1

**Published:** 2026-05-14

**Authors:** Kai PURNHAGEN, Detlef Bartsch, Kolja Eckermann, Aleksandra Hubar-Kołodziejczyk, Jörn Lämke, Steffen Pallarz, Justus Wesseler

**Affiliations:** 1 University of Bayreuth, Bayreuth, Bavaria, Germany; 2 Institute of Veterinary Biochemistry, University of Berlin, Berlin, Germany; 3 Bundesamt fur Verbraucherschutz und Lebensmittelsicherheit Berlin Mitte, Berlin, Berlin, Germany; 4 Wageningen University & Research, Wageningen, Gelderland, The Netherlands

**Keywords:** New Genomic Techniques (NGTs), genome editing, Detection, Identification and Quantification (DIQ), single nucleotide variants (SNVs)

## Abstract

For the purposes of GMO authorisation, applicants are required to submit validated methods for the detection, identification and quantification (DIQ). For classical transgenic organisms, this requirement has proven workable. For products derived from New Genomic Techniques (NGTs), specifically those introducing small insertions and deletions (InDels) without foreign genetic material, it has not. NGT-induced genomic changes are frequently indistinguishable from naturally occurring mutations, making identification legally required but technically unachievable with currently available analytical tools. This open letter examines whether recently proposed DIQ methodologies, including genomic fingerprinting approaches based on single nucleotide variant (SNV) profiling, can bridge this gap. We find that while such approaches advance detection capabilities, they fall short of the identification and quantification standards demanded by EU law, for reasons such as the absence of universal pangenome datasets, the complexity of polyploid crop genomes, the loss of fingerprint integrity through elite line crossing, and the dependence of recombination rates on multiple species-specific factors. The mismatch between legal requirements and scientific feasibility has consequences for authorisation procedures, supply chain compliance, organic farming integrity, and international trade. We conclude that no purely technical solution is forthcoming and that legislative change is necessary. Aligning the EU’s evidentiary standards with technical reality is a precondition for a functional and enforceable GMO authorisation system.

## Legal requirements

The EU’s GMO framework sits among the most demanding regulatory regimes for biotechnology products in the world, ^1^ making most products manufactured with New Genomic Techniques (NGTs) ^2^ subject to it. ^3^ This article addresses NGTs that create mutation(s) without insertion of foreign genetic material, specifically small insertions and deletions (InDels). ^4^ Under EU GMO law, detection, identification and quantification (DIQ) methods are required to receive an authorisation for the placing on the market (in particular import) and cultivation of GMOs and GM food and feed. ^5^ Absent reliable DIQ methods, effective enforcement of EU GMO and non-GMO rules cannot be achieved in practice. ^6, 7^ This has significant implications for (1) submission for authorisation for release into the environment and/or for import and processing, (2) international trade with third countries, where not all GMOs are subject to labelling, authorisation or registration requirements, (3) the restriction or prohibition of GMO cultivation to be enforced by Member States under Directive (EU) 2015/412, (4) the exclusion of GMOs by producers in organic production, (5) the provision of freedom of choice for consumers and (6) sustainable agriculture development in the EU.

EU law mandates that the submitted methods must be capable of detecting, identifying and quantifying a specific genetic modification. ^8^ Detection establishes the presence or absence of a genomic change (also at phenotypic level). ^9, 10^ Identification then attributes that change to a particular modification event, distinguishing between spontaneous mutation and deliberate technical intervention. ^11^ Quantification assigns a measured mass fraction to a defined GM event, enabling assessment against the labelling thresholds set by EU law. ^12^ Even with validated methods for detection and quantification, identifying NGT events - in contrast to the identification of classical GMOs - remains challenging, if not impossible. ^13, 14, 15^ One could claim that in practice detection methods for classical GMOs may indirectly be used also for identification, as the genetic changes in classical GMOs containing transgenic constructs are unique for the event and generally the result of technical intervention. However, small genomic edits in NGT plants can be identical to modifications that could occur naturally or through classical breeding techniques, ^9, 13, 14^ making the establishment of causal links between detection and identification of an NGT-derived line highly challenging or even impossible from a biological point of view. Furthermore, it is questionable if an interpretation of a detection based on likelihoods would serve the legal requirements for establishing causal links. In the EU, DIQ methods are available for products that have been authorized, as they are a legal requirement for receiving authorisation. In contrast, DIQ methods for unauthorised GMOs need to be developed by the public and/or private sector. Food business operators are obliged to do so to operationalise their legal duty to develop control mechanisms in order to verify their compliance with GMO food laws. Existing control mechanisms for unauthorized GMOs are largely designed and developed based on publicly available information. Obtaining positive materials necessary for method validation is often difficult and depends on the readiness of the manufacturer to provide those.

Testing for unauthorized GMOs remains at the discretion of the responsible national control agencies. Traders and processors of food products require, as part of their contractual obligations, declarations regarding the GMO status of the product from downstream market participants. Hence, within the agriculture and food supply chain, these requirements trace back to the producer. In case of crops, GMOs approved for cultivation in a country outside the EU but not within the EU might reduce the production choices of the farmer outside of the EU if the product produced on her/his farm is intended to enter the EU market. They also affect the organisation of international agriculture and food supply chains. They add due to the increased complexity of organising agriculture and food supply chains, which, in the end, have to be paid for by the final consumer through higher food prices. ^16^

EU authorisation operates through two parallel tracks ^17, 18^: deliberate release and market placement fall under Directive 2001/18/EC, while food and feed authorisation is governed by Regulation 1829/2003 and its implementing regulations. This includes GM animals and microorganisms. The majority of applications pertain to GM food and feed of plant origin; hence the GMO Regulation 1829/2003 is more significant in practice. ^19^ However, product authorisation can be sought simultaneously, and liability has been shown to appear across authorisation paths. ^7^ Consequently, the individual legal requirements for authorisation should be considered together.

DIQ methods in food and feed are governed by Commission Implementing Regulation (EU) No 503/2013 ^20^ (the GMO Food Authorisation Regulation) and Commission Regulation (EC) No 641/2004. ^21^ Cultivation and placing on the market is governed by the GMO Directive, read together with national implementing legislation. The “one door one key principle” connects both regimes.

For GM higher plants, the GMO Directive requires a description of the detection and identification techniques. ^22^ DIQ methods must not be considered confidential (Article 25 of the GMO Directive); only detailed DNA sequence information fulfils this requirement, which in turn requires the use of analytical methods. ^7^ Implementing Member States cannot and do not deviate from these requirements. ^10^

The authorisation procedure outlined in the GMO Regulation concerning food and feed likewise requires the submission of methods. Article 8 of the GMO Food Authorisation Regulation and Commission Regulation (EC) No 641/2004 are framed exclusively around analytical methods, ^19^ with PCR-based ^7^ approaches at the core. Critically, unlike other elements of the authorisation dossier, no exemption or derogation is available for DIQ requirements: the legislator treated these as a strict, non-negotiable element of the enforcement regime. Their categorisation as part of the enforcement process also justifies such a strict legal regime. ^19^ Legal limits to analytical DIQ methods is supported by the practical requirements applicants and laboratories validating the methods face in the authorisation procedure and is shared by the European Network of GMO Laboratories (ENGL). ^19^

## Compliance of proposed DIQ methods for NGTs with legal requirements

One publication ^23^ advertised a basic breakthrough in DIQ methodology. While the proposed methodology is promising for the detection “D” part, major limitations remain for the “I & Q” parts due to the following shortcomings:

- A fundamental prerequisite for the design of fingerprint, i.e. a set of single nucleotide variants (SNVs) which unambiguously identifies a variety line, is the availability of a robust and extensive genomic data set, which includes numerous high-quality reference genomes from a large variety of lines within a species (pangenome). While such data sets may exist for some species, e.g. rice, they are not yet attainable for many other species.
- The chosen example, rice, being a diploid organism, possesses a relatively simple genomic structure, which makes the approach more feasible for this species. However, many other crop species are polyploid and have more complex genomes. Although this complicates the method’s application, future advancements in fingerprint development could potentially alleviate this issue to some extent.
- Most importantly, the method does not provide identification. Rather, it creates a fingerprint to trace the origin line and then detects GE-related SNVs within this line. As there is no individual fingerprint for the actual NGT organism, the method does not allow for the differentiation between a NGT-organism and one with the same background where the mutation occurred naturally as there are no links between the fingerprint and the additionally interrogated SNV.
- Moreover, the identification process described in the paper is not a true identification of the origin line but rather an elimination of other well-sequenced lines. Therefore, unless all varieties of a species are used to create the fingerprint, it cannot be considered unambiguous.
- Whereas the approach chosen might be amendable to genetically homogeneous materials, it falls short if mixtures of lines need to be analyzed, which is the most realistic scenario for GMO analyses.
- Another fundamental problem of the analytical strategy arises from the common practice of plant breeders to cross the developed events (modifications) from the developing line into their many different geographically and climatically specific elite lines, resulting in a loss of the fingerprint and a strongly altered SNV profile for each of these lines. Therefore, a new fingerprint would have to be established for each of these elite lines carrying the event, again requiring the supply of the respective positive material. For comparison: there are more than 100 different lines in Europe for the EU-approved MON810 maize event (Bayer Crop Science) alone. It would therefore be necessary for the fingerprint-specific SNVs to be genetically linked to the edited locus, ensuring their preservation in subsequent generations and crossbreeds. ^14, 15^
  However, since the recombination rate depends on various factors, including the total genome size, methylation, the occurrence of recombination hotspots, and imbreeding, ^24, 25, 26, 27^ it is not feasible to determine a general or species-specific value for a genomic distance or centimorgans in order to establish a generation- and lineage-spanning fingerprint for the event.
- In fact, the proposed method does not provide better “D&I” abilities than a WGS- or even amplicon-sequencing based approach paired with bioinformatic analyses (i.e. determine the variety by means of blast or alignment against references followed by SNV calling). What it does is offer a more targeted approach allowing for the design of PCR based detection methods targeting the fingerprint and the GE SNV, which does have advantages for routine analyses in authorisation settings, but it does not solve any of the D&I issues previously discussed.

## Conclusion

The EU’s regulatory architecture for GMOs was built on the premise that every authorised product can be uniquely detected, attributed to a specific modification event and quantified with analytical certainty. For classical transgenic organisms, this premise has largely held. For NGT-derived products producing small, locus-specific alterations, it no longer does. As molecular breeding technologies advance, the gap between what EU law requires and what science can deliver has widened to the point of structural incompatibility.

The central issue is not detection but identification. Although genomic differences can be observed with increasingly sensitive (sequencing) technologies, the legal requirement to identify whether a mutation arose through technical intervention (and to link it to a defined event in the sense of attribution) cannot be met when NGT-induced changes are indistinguishable from naturally occurring variants. No analytical refinement, whether whole-genome sequencing or targeted “fingerprinting,” can transcend this biological limitation. These methods may streamline detection within well-characterized species, but they do not provide the unambiguous identification or event-specific quantification that EU law demands.

This mismatch has cascading consequences. Enforcement becomes inconsistent, authorisation dossiers become legally precarious, and supply chains (both within and beyond the EU) must absorb the costs of compliance systems that do not reliably discriminate the products they are designed to police. Farmers, traders and regulators alike are left navigating obligations that cannot be fulfilled with the tools currently available. As international breeding and trade systems continue to integrate genome editing, the EU risks isolating itself behind an evidentiary standard that no longer corresponds to biological reality.

If DIQ requirements are to remain central to the EU’s governance of genomic technologies, the legislation itself must evolve. A framework predicated on event-specific traceability is ill-suited for mutation-based NGTs and will only become more strained as these tools proliferate. Aligning regulatory expectations with scientific feasibility (whether through revising definitions, adapting evidentiary standards or developing alternative risk-based approaches) is now essential. Without legislative change, the EU’s authorisation system will remain scientifically untenable, practically unenforceable and increasingly disconnected from the innovation landscape it seeks to regulate.

## Disclaimer

The views expressed in this article are those of the author(s). Publication in Open Research Europe does not imply endorsement of the European Commission.

## Ethics and consent

Ethical approval and consent were not required for this study.
