# Supplementary material for: Detection, identification and quantification of NGTs in EU authorization procedures – No solution without legislative change
Source: Open Res Eur. 2026 May 14;6:168. [Version 1] doi: 10.12688/openreseurope.23629.1 (PMC13261188; doi:10.12688/openreseurope.23629.1)
Supplement: Peer review history

# Reviewer report: Reviewer response for version 1

**DOI:** 10.21956/openreseurope.25583.r74841

**Published:** 2026-06-11

**Authors:** Alexios N Polidoros

**Affiliations:** 1 Aristotle University of Thessaloniki, Thessaloniki, Greece

The **open letter** manuscript ( https://doi.org/10.12688/openreseurope.23629.1) attempts to address the challenges of **detection, identification, and quantification** of plants obtained through New Genomic Techniques (NGTs) within EU authorization procedures. The authors argue, based on experiences with GMOs, that effective traceability and regulatory oversight of NGT-derived products require significant legislative changes to ensure transparency for producers and consumers throughout the product lifecycle.

 While the topic is highly relevant to ongoing debates in EU agricultural biotechnology policy, the letter requires **major revisions** before it can be considered suitable for indexing.

 Major weaknesses:

1. **Outdated content and failure to reflect current regulatory developments:** The core premise—that “there should be no solution without legislative change” and that NGTs must be treated similarly to GMOs—does not accurately represent the evolving EU framework. The authors overlook the provisional political agreement reached on 3 December 2025 between the European Parliament and Council ( https://ec.europa.eu/commission/presscorner/detail/de/ip_25_2912). This agreement introduces a differentiated, two-tier system:
  - **NGT-1 plants and products** (those indistinguishable from conventional plants) will follow a product-based approach, exempt from GMO regulations.
  - **NGT-2 plants and products** will remain subject to existing GMO rules.

Subsequent steps include the ENVI Committee approval (28 January 2026) ( ref), the Council endorsement (21 April 2026) ( ref), and the formal adoption by the Council (May 2026) ( ref), with publication expected in 2026 and full application around mid-2028 after a transitional period.

1. **Lack of balance and global context:** The letter adopts a process-based regulatory stance for all NGTs, overlooking the EU’s partial transition toward product-based regulation for NGT-1, which aligns with approaches taken by countries such as the USA, Canada, the UK, Australia, Japan, and others. Additionally, it downplays the potential benefits of NGTs ( ref) for climate resilience, sustainability, and competitiveness, as emphasized in recent statements from European agri-food organizations. For instance, on May 4, 2026, a coalition of 31 European agri-food value chain organizations issued a joint statement advocating for the prompt adoption of the NGT regulation without further amendments ( ref). Furthermore, in a letter dated May 15, 2026, four prominent European plant biotechnology associations urged the members of the ENVI Committee to swiftly approve a pending compromise regulation on NGTs ( ref). This letter was endorsed by the French Association of Plant Biotechnologies (AFBV), Forum Grüne Vernunft (FGV), the Society for Plant Biotechnology (GfPB), and the Genomics and Genetic Engineering Research Circle (WGG).
2. **Incomplete discussion of practical implications:** With several NGT products nearing market entry ( ref), the letter should better inform stakeholders by presenting the full, up-to-date landscape rather than an outdated, GMO-centric view.

Recommendations for major revision:

1. Update the entire text to incorporate the 2025–2026 legislative timeline and the NGT-1 vs NGT-2 distinction.
2. Revise the central argument to differentiate regulatory requirements between categories.
3. Add a balanced discussion of benefits (e.g., reduced inputs, sustainability) alongside detection challenges for NGT-1 products.
4. Include recent references to industry coalition statements and implementation timelines.
5. Strengthen the letter with clearer structure, updated citations, and acknowledgment of the transitional period toward 2028 applicability.
6. Modify the title accordingly to better reflect the message after the proposed changes.

In its current form, the open letter presents an incomplete and partially obsolete picture of the EU NGT regulatory situation. A **major revision** is necessary to ensure scientific and policy accuracy. After addressing these issues, particularly by incorporating developments post-December 2025, the letter could make a valuable contribution to the discourse on NGT governance in Europe.

Where applicable, are recommendations and next steps explained clearly for others to follow? (Please consider whether others in the research community would be able to implement guidelines or recommendations and/or constructively engage in the debate)

Partly

Does the article adequately reference differing views and opinions?

No

Are all factual statements correct, and are statements and arguments made adequately supported by citations?

Partly

Is the rationale for the Open Letter provided in sufficient detail? (Please consider whether existing challenges in the field are outlined clearly and whether the purpose of the letter is explained)

Yes

Is the Open Letter written in accessible language? (Please consider whether all subject-specific terms, concepts and abbreviations are explained)

Yes

Reviewer Expertise:

Plant Molecular Biology, Genetics , Biotechnology, Breeding

I confirm that I have read this submission and believe that I have an appropriate level of expertise to confirm that it is of an acceptable scientific standard, however I have significant reservations, as outlined above.

# Reviewer report: Reviewer response for version 1

**DOI:** 10.21956/openreseurope.25583.r74849

**Published:** 2026-06-09

**Authors:** Federica DeMaria

**Affiliations:** 1 Council for Agricultural Research and Economics (CREA), Rome, Italy

The manuscript addresses a major unresolved conflict between EU agricultural law, supply chain economics, and innovation policy. It examines the EU GMO framework, which requires Detection, Identification, and Quantification (DIQ) for market authorization under Directive 2001/18/EC and Regulation (EC) No 1829/2003. Economically, this requirement aims to reduce information asymmetry and protect consumer choice. While suitable for conventional transgenic products, the paper argues that it becomes problematic for New Genomic Techniques (NGTs), especially simple edits without foreign DNA. These edits are molecularly indistinguishable from natural mutations and therefore cannot be reliably detected by control authorities. The manuscript also critiques a recent proposed technical solution, showing that it fails because breeding processes break the traceable genetic “fingerprint.” As a result, the current framework risks generating market fragmentation, higher compliance and transaction costs, and trade disruption. The authors conclude that no workable technical fix is likely to emerge. Therefore, a legislative revision is necessary to restore regulatory certainty and market efficiency.

**Economic & Policy Critique**: The manuscript clearly highlights the risks this analytical deadlock poses to international trade, supply chain compliance, and the organic food market. However, its rationale is not fully aligned with the institutional and policy context of 2026. The authors state that “legislative change is necessary,” overlooking that EU institutions are already finalizing a major regulatory overhaul through the new NGT Regulation, which classifies NGTs into two categories (NGT1 and NGT2). To strengthen the argument, the paper should emphasize the core regulatory and economic paradox of this transition: the EU plans to exclude NGT1 products from organic farming while also exempting them from mandatory labeling and DIQ testing. This creates an impossible compliance burden and significant negative externalities for the organic sector, which must ensure the absence of NGTs without having the analytical tools to verify it.

**Economic & Policy Critique**:  The paper models the regulatory dilemma as a static technical failure rather than an active conflict between competing regulatory philosophies and economic interests. To be robust, it must reference two opposing viewpoints: a) the Product-Based / Innovation View represented by the agro-biotech industry and seed developers; b) the Process-Based / Precautionary View: Represented by environmental NGOs and the organic food sector.

**Economic & Policy Critique**: The economic and institutional reasoning is highly accurate, but the text lacks the foundational institutional citations required to make its legal-economic arguments, for instance, when stating that NGTs fall under strict GMO law, the authors must cite the landmark CJEU Case C-528/16 (2018 ruling), which is the institutional shock that legally triggered these market frictions; furthermore, when arguing that verification is an impossible standard for enforcement agencies, they should cite the European Network of GMO Laboratories (ENGL) 2019 report, which provides the official institutional baseline proving that laboratory enforcement of unauthorized simple edits is unfeasible.

**Economic & Policy Critique:** While the authors accurately call for a legislative evolution, their recommendations are too abstract for policy implementation. The authors need to move beyond stating *what* is broken and provide concrete, actionable policy pathways regarding how the EU can shift from costly analytical verification to institutional workarounds.

 Finally, regarding the **conclusion**, I would suggest expanding them with this economically grounded framework

Where applicable, are recommendations and next steps explained clearly for others to follow? (Please consider whether others in the research community would be able to implement guidelines or recommendations and/or constructively engage in the debate)

Partly

Does the article adequately reference differing views and opinions?

Partly

Are all factual statements correct, and are statements and arguments made adequately supported by citations?

Partly

Is the rationale for the Open Letter provided in sufficient detail? (Please consider whether existing challenges in the field are outlined clearly and whether the purpose of the letter is explained)

Partly

Is the Open Letter written in accessible language? (Please consider whether all subject-specific terms, concepts and abbreviations are explained)

Partly

Reviewer Expertise:

Applied Economics Agricultural and Food Economics Regulatory Impact AnalysisSupply Chain Economics

I confirm that I have read this submission and believe that I have an appropriate level of expertise to confirm that it is of an acceptable scientific standard, however I have significant reservations, as outlined above.

# Reviewer report: Reviewer response for version 1

**DOI:** 10.21956/openreseurope.25583.r74410

**Published:** 2026-05-20

**Authors:** Juan Antonio Vives Valles

**Affiliations:** 1 University of the Balearic Islands, Palma, Spain

The open letter focuses on one of the most legally significant questions in the field of GM crops, food, and feed in the EU, the “detection, identification and quantification[ in the light] of NGTs”.

 The difficulty of detection is also probably one of the most overused arguments in favour of deregulating NGTs and, despite its technical basis and significant implications, also one of the most controversial (albeit persuasive) arguments. It assumes, from the outset, widespread and systematic non-compliance on the part of breeders, and cunningly presents deregulation as the only possible solution.

 The open letter shows that the underlying problems remain unresolved.

 In view of potential legislative developments in relation to the key issues raised in the letter, it is suggested that due attention be paid to the wide array of nuances inherent in the overly broad and complex notion of *NGT plants*.

Where applicable, are recommendations and next steps explained clearly for others to follow? (Please consider whether others in the research community would be able to implement guidelines or recommendations and/or constructively engage in the debate)

Yes

Does the article adequately reference differing views and opinions?

Yes

Are all factual statements correct, and are statements and arguments made adequately supported by citations?

Yes

Is the rationale for the Open Letter provided in sufficient detail? (Please consider whether existing challenges in the field are outlined clearly and whether the purpose of the letter is explained)

Yes

Is the Open Letter written in accessible language? (Please consider whether all subject-specific terms, concepts and abbreviations are explained)

Yes

Reviewer Expertise:

Intellectual Property and Biosafety Law...

I confirm that I have read this submission and believe that I have an appropriate level of expertise to confirm that it is of an acceptable scientific standard.
